# Theoretically-informed vs standard cover letter to improve participant response to mailed questionnaire: results of an embedded randomised retention trial

**DOI:** 10.1186/s13063-024-08565-0

**Published:** 2024-11-14

**Authors:** Colin C. Everett, Sarah T. Brown, Joanna L. Dennett, Howard Collier, Claire L. Davies, Frances Game, E Andrea Nelson

**Affiliations:** 1 Clinical Trials Research Unit, Leeds Institute of Clinical Trials Research, University of Leeds, Leeds, UK; 2 University Hospitals of Derby and Burton NHS Foundation Trust, Derby, Derbyshire UK; 3 Glasgow Caledonian University, Glasgow, Scotland, UK

**Keywords:** SWAT, Study within a trial, Retention methods, Embedded randomised controlled trial, Surveys and questionnaires, Behavioural change theory, Diabetic foot, Follow-up studies, Quality of life, Outcome assessment, Health care

## Abstract

**Background:**

Participant non-response is a source of bias in all research, especially in randomised controlled trials. Participants followed up remotely can have high non-response rates. Four such trials have been conducted of a cover letter with content informed by behaviour change theory to overcome hypothesised barriers to responding to a mailed questionnaire. Pooled results to date have suggested further research to be worthwhile. We conducted an embedded randomised study within a trial of such cover letters in the hope that we would improve response rates to our postal quality of life questionnaires.

**Methods:**

One hundred forty-eight participants in the CODIFI2 diabetic foot ulcer sampling trial were randomised 1:1 to receive one of two different cover letters at follow-up timepoints: either a standard cover letter accompanying their postal follow-up questionnaires or to an ‘enhanced’ (theory-informed) cover letter. Questionnaires were mailed at 39, 52 and (for some participants) 104 weeks post randomisation. Outcome measures were response to mailing at each timepoint. Analysis was restricted to those for whom a questionnaire and letter was issued. Owing to limited recruitment, a reduced analysis plan, comprising solely observed response rates and 95% confidence intervals for difference in response rates was followed. Post hoc, we added our week 52 results to an already-published meta-analysis.

**Results:**

Sixty-seven out of 74 enhanced cover letter group (Enhanced) and 67/74 standard cover letter group (Standard) participants who had not already died or withdrawn were sent their first mailing at 39 weeks. The 39-week response rates were 47/67 (70.1%) and 39/67 (58.2%) for Enhanced and Standard participants, respectively. At week 52, the response rates were 45/64 (70.3%) and 35/63 (55.6%) for Enhanced and Standard participants, respectively. At week 104, the response rates were 24/33 (72.7%) and 19/33 (57.6%) for the Enhanced and Standard participants, respectively. Adding our week 52 results to a published meta-analysis increased the pooled estimate of differences in response rates to 0.04 (− 0.01 to 0.09) favouring enhanced letters.

**Conclusion:**

While this embedded randomised controlled trial observed greater response rates at all times among those randomised to the enhanced letter, the reduced sample size meant that these results are imprecise.

**Trial registration:**

ISRCTN registry ISRCTN74929588. Registered on 5 March 2019.

**Supplementary Information:**

The online version contains supplementary material available at 10.1186/s13063-024-08565-0.

## Introduction

If participants in randomised controlled trials (RCTs) do not provide outcome data at the expected times, then any analyses undertaken may become unreliable. Attrition leads to a loss of outcome data, which leads to both a loss of statistical power to detect clinically relevant differences, and more importantly, potentially biased estimates of the treatment effect [1]. Statistical methods exist for analysing outcome data in the presence of missingness, but must make unverifiable assumptions about the unobserved data [2–4]. Consequently, it is always preferable to design and conduct a study to maximise the chance of observing the expected outcome data. This is reflected in the findings of the PRioRiTy II study (Prioritising Retention in Randomised Trials [5]), reporting that one of the top ten questions for clinical trial conduct concerned ‘[…] the best ways to encourage trial participants to complete the tasks (e.g. attend follow-up visits, complete questionnaires) required by the trial?’.

Mailed questionnaires are one possible method to collect outcome data. These can be more convenient to a participant than requiring them to attend an in-person clinic appointment and hence can address a potential barrier to recruitment and retention to RCTs—which may be related to participant characteristics—and hence external validity [6, 7]. Unfortunately, mailed questionnaires can suffer from low response rates even over short durations. For example, in a 2018 RCT of different response options to a survey mailed to parents of patients in a childhood diabetes registry, Bjertnaes et al. [8] reported response rates between 41.6% and 62.4% for a survey administered at a single timepoint. Despite the promise of valid results if unobserved values can be predicted by other observed baseline covariates and outcome data (the ‘missing at random’ assumption [2]), examples such as this demonstrate the need to design the process for collecting outcome data remotely (including via mailed questionnaires) so as to encourage responses.

A 2009 Cochrane Review [9] identified 481 eligible trials evaluating 121 different interventions aiming to improve response rates to mailed or electronic questionnaires. A more specific Cochrane Review by Gillies et al. in 2021, tailored to improving retention in RCTs [10], identified 69 studies assessing 52 interventions targeting a variety of follow-up aspects, including mailed questionnaires. This latter review found that evidence supporting the proposed interventions was generally uncertain, per GRADE (Grading of Recommendations, Assessment, Development and Evaluations) criteria.

One intervention that has been previously trialled is an enhanced cover letter, with content informed by behavioural change theory [11, 12]. The iQUAD (Improving Quality of Dentistry) trial authors who devised the original intervention identified several potential barriers to a participant responding to a questionnaire and tailored a cover letter to address these barriers by incorporating wording in line with behaviour change techniques, such as stressing the degree of support for the trial and its importance [11]. While the pooled evidence from four RCTs embedded within existing research [10] hinted at a benefit to these enhanced letters over a ‘standard’ comparator letter, the 95% confidence interval for the pooled effect included zero. We decided to undertake a replication of this embedded study within a trial (SWAT) in a recent RCT, with the aim being to estimate the extent to which the theoretically informed cover letter (adapted to our own RCT) improved response rates (or did not) to mailed questionnaires.

## Methods

### Host RCT and SWAT

The CODIFI2 (Concordance in Diabetic Foot Infection 2) multicentre randomised controlled trial (ISRCTN74929588) included an embedded multicentre, prospective, two-arm parallel-group randomised SWAT to evaluate an intervention to improve postal questionnaire response rates. The main CODIFI2 RCT recruited people with diabetes and diabetes-related foot ulcers (DFU) that were suspected to be infected, from specialist centres in England from May 2019 to April 2022. The aim of CODIFI2 was to determine whether sampling potentially infected DFUs by swab sampling or by tissue curettage (to determine appropriate antibiotic usage) would result in faster DFU healing. Full eligibility criteria for CODIFI2—and by extension this SWAT—are included as Supplementary Appendix 1. No additional eligibility criteria applied to this SWAT: all participants eligible for the CODIFI2 main RCT were eligible to be randomised in the SWAT.

### Ethical approval and informed consent

This SWAT was incorporated within CODIFI2’s ethically approved protocol from the outset, and ethical approval for the study as a whole was obtained from West of Scotland REC 3 (Research Ethics Committee, Ref 18/WS/0235). The CODIFI2 protocol, which includes the details of this SWAT, is available from the funder at [13]. Participants were informed in the participant information leaflet that there would be a trial of communication methods but were not given further details.

### Interventions

At weeks 39, 52 and 104 (the latter for the earliest CODIFI2 recruits only), participants were mailed questionnaire booklets and a cover letter, according to their randomised allocation. Cover letters were automatically generated by means of database reports, which generated the correct letter for each participant according to their randomised allocation, the timepoint of interest, expected follow-up duration and (for week 52) receipt of a response at week 39. For brevity, this article generally refers to the ‘Standard’ and ‘Enhanced’ cover letters in this article: the ‘enhanced’ cover letter being based on the theoretically informed letter identified on the SWAT repository in the SWAT24 entry [14] and provided by the Trial Forge evidence pack [15]. Samples of the enhanced and standard cover letters (and electronic reminders) used in our replication of this SWAT are included as Supplementary Appendices 2 and 3.

A participant randomised to the standard letter arm in the SWAT received the standard cover letter, which merely reminded the participant of their involvement requesting the completion and return of their booklet, and of a participant-completed antibiotic diary. A participant randomised to the enhanced letter arm received a letter with content hypothesised to address barriers to non-response. This letter was mostly drafted along the lines of that used in the iQUAD RCT [12] but tailored to the requirements of CODIFI2’s design and accounted for feedback from a participant and public involvement (PPI) group, which we describe in a subsequent paragraph. All letters (except the reminder) included the same photograph of the same CODIFI2 co-chief investigator and included contact details for a member of the CTRU data management team to provide further assistance if this was required.

All participants were sent identical reminder letters in case of non-response. While most participants opted for electronic reminders by email and/or SMS, we also issued mailed reminder letters. Reminder letters (which included a fresh questionnaire booklet) were similar to the Standard group letter in style and tone, so as to test the effect of changing the initial mailing (the letter and electronic forms of the reminders are included in Supplementary Appendices 2 and 3). At week 52, participants received slightly different cover letters depending on whether they were intended to be followed up to 104 weeks. At their final postal follow-up, whether at 52 or 104 weeks, all letters thanked the participant for their involvement.

### Randomisation and blinding

Eligible and consenting participants were randomised to the host RCT arms (swab or tissue sampling of the index DFU) by site research staff via a 24 h automated interactive central randomisation system operated and maintained at the University of Leeds CTRU (Clinical Trials Research Unit). Immediately afterwards, the system randomised participants in a 1:1 allocation ratio to receive one style of cover letter (standard vs enhanced) consistently at each of their postal follow-up timepoints at weeks 39, 52 and 104 post randomisation, for those participants randomised in the first year of CODIFI2’s recruitment. SWAT randomisation was by minimisation with a random element (75%), with the minimisation factors being age (65 or younger vs 66 or older), sex, randomising centre and the main CODIFI2 randomised allocation (swab sampling vs tissue sampling). We balanced for age and sex since we a priori considered these factors predictive of response. We balanced for centre, as the site may have acted as a proxy for other factors that predicted response (such as quality of care from site staff). Finally, we balanced for the main site randomised arm, so as to ensure that any intervention effects were not accidentally confounded with the randomised letter group effects.

Neither site staff (recruiting, treating or assessing study outcomes) nor participants were informed of their cover letter allocation at the moment of randomisation. CTRU staff involved in generating cover letters and entering returned data were not blind to randomised outcome. Participants did not receive their first cover letter until their first postal follow-up, 39 weeks post randomisation. Participants were not informed that their mailings were chosen as one from two possible style of mailings, that the choice of mailing style was made at random, nor that the style of mailing would be the same at each of that participant’s mailed follow-up timepoints.

### Statistical methods

The SWAT’s original sample size was determined by that of the main CODIFI2 study. For information, we produced a sample size calculation to illustrate the potential power to the study to detect an effect of enhanced cover letters on response rates. With the intended 730 recruited participants, the study would have 80% power to detect an absolute difference of 10 percentage points in response rates with a two-sided 5% significance test, allowing for analysis of all timepoints as repeated measurements clustered within participant (assuming intra cluster correlation of 0.7) and a control group response rate of 50%, and 10% loss to follow-up before week 39. No interim analyses of the SWAT were planned or undertaken.

The outcome measures for the SWAT were the proportions of mailings issued for which we received the questionnaire in return for each of the three timepoints in isolation. The analysis populations for each timepoint were defined as the participants randomised for whom a questionnaire was issued: any randomised participants dying, withdrawing from postal questionnaire follow-up or completing follow-up prior to a timepoint were excluded from the analysis for that timepoint, as were participants for whom questionnaires were not sent. We estimated for each group the proportion of participants returning a questionnaire (regardless of whether they were returned complete or in any usable state for host RCT outcome analyses) and the absolute differences in proportions returning. Confidence intervals (CI) for proportions and the absolute differences in proportions were by the Clopper–Pearson Exact method for single proportions [16] and the Santner–Snell [17] exact interval for differences in proportions. No hypothesis testing was undertaken. These analyses were performed using SAS 9.4.

Post hoc, we updated the meta-analysis published by Gillies et al. [10] to include the 52-week CODIFI2 findings. This was an exploratory post hoc analysis: we did not assess risk of bias or investigate the heterogeneity of interventions and outcome measures prior to adding our findings to the analysis. Taking the published numbers of events reported in analysis 43 of this review, we also included the numbers from our study that responded and analysed for the two cover letter groups at 52 weeks. This timepoint was chosen as it represented the expected minimum follow-up for all CODIFI2 participants. Our meta-analysis of risk differences used the DerSimonian–Laird estimator for between study variance and Mantel–Haenszel Estimates in the calculation of variance and Cochran’s *Q* statistic. We verified that the analysis choices were aligned with those previously published in Gillies et al. before proceeding to our updated analysis. The post hoc meta-analysis update used R 4.2.3 [18] and the meta package v7.0.0 [19].

The host trial collected data on harms for the study as a whole (host trial and substudies) with provision for adverse events not related to the main trial interventions or underlying comorbidities to be reported separately. Details of adverse events reported across the CODIFI2 RCT as a whole will be reported with the main study results.

#### Participant and public involvement

The CODIFI2 host RCT included lay members on the Trial Management Group and Trial Steering Committee. There was no dedicated PPI representative for the SWAT. In tailoring the intervention to our study, we presented initial drafts of Enhanced and Standard cover letters to an in-person meeting of the LADDER (Lay ADvice on Diabetes and Endocrine Research) PPI group on 12 September 2018, based at Royal Hallamshire Hospital, Sheffield. We asked for opinions on the actual conduct of the SWAT and on the content of the letters themselves. Following the group’s support for the SWAT and feedback on changes to the letters, we made some amendments to the Enhanced letters. Specifically, we omitted a detailed action plan for completion (owing to length), removed all references to the questionnaires being short (following LADDER members’ scepticism) and did not have local site staff signatures owing to difficulties in implementation. We also added to enhanced Week 52 letters a sentence thanking participants for responding at Week 39 where this had happened.

### Changes to study design and outcomes

CODIFI2 was closed to recruitment and follow-up before reaching its planned sample size, and a truncated analysis plan was agreed with the funder. Consequently, the SWAT also closed to recruitment early. We originally intended to perform exploratory analyses estimating differences in times to return of questionnaires, but this was complicated by the COVID19 (Coronavirus Disease 2019) pandemic. Questionnaires were customarily date stamped on arrival at the CTRU, but national lockdowns meant that the date stamps reflect dates that offices were open to process mail, rather than actual dates of receipt. Consequently, we did not perform any analyses of time to return.

## Results

**Fig. 1:**
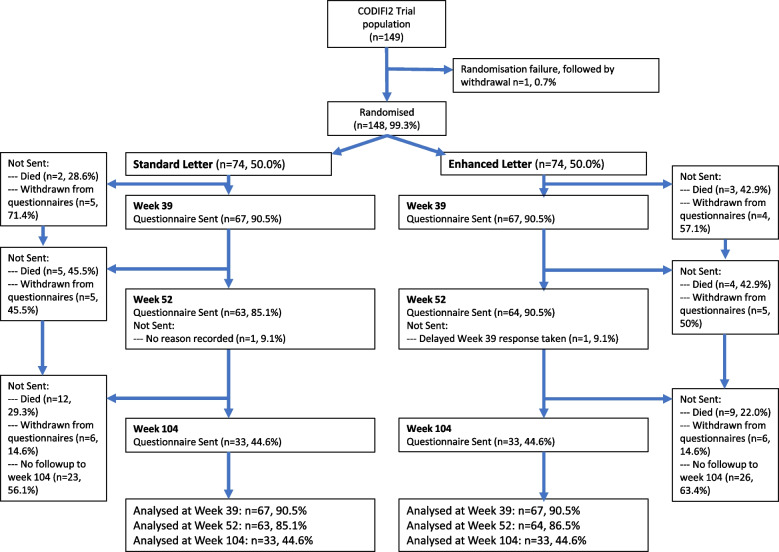
Participant flow diagram for cover letter SWAT

Between 7 May 2019 and 3 May 2022 denoting first and final recruitment, 148 of 149 CODIFI2 participants were randomly assigned to a cover letter group. The follow-up period ended 52 weeks after the final participant’s recruitment. One participant was not randomised owing to a randomisation system failure and subsequent withdrawal prior to week 39 timepoint. Figure 1 illustrates the participant flow through the two arms, including the numbers still in the study at each of the follow-up timepoints.

The baseline characteristics of the 74 standard letter and 74 enhanced theory-informed letter participants are summarised in Table 1. The two groups were similar in most respects, the mean age was 62.8 years (standard deviation 12.55, range 31 to 93), 122/148 (82.4%) were male and 143/148 (96.6%) were of white ethnicity; 130/148 (87.8%) of participants had type II diabetes, with median (interquartile range) diabetes duration 15.5 years (10 to 21.5 years) and 96/148 (64.9%) taking some oral hypoglycaemic agent and 83/148 (56.1%) taking insulin. It appeared that participants randomised to the standard cover letter were more likely to report no problems with mobility (29.7% vs 13.5%) and anxiety/depression (60.8% vs 50.0%) on the baseline Euroqol EQ5D-3L questionnaire than the enhanced letter.

**Table 1:** Baseline characteristics of all randomised SWAT participants

	**Standard letter (*n* = 74)**	**Enhanced letter (*n* = 74)**	**Total (*n* = 148)**
Age, years			
Mean (SD)	63.1 (13.06)	62.4 (12.10)	62.8 (12.55)
Range	(31 to 93)	(31 to 92)	(31 to 93)
Gender			
Male	62 (83.8%)	60 (81.1%)	122 (82.4%)
Female	12 (16.2%)	14 (18.9%)	26 (17.6%)
Ethnicity			
White	71 (95.9%)	72 (97.3%)	143 (96.6%)
Mixed—White and Black Caribbean	-	1 (1.4%)	1 (0.7%)
Asian—Pakistani	-	1 (1.4%)	1 (0.7%)
Other Asian background	1 (1.4%)	-	1 (0.7%)
Black—African	2 (2.7%)	-	2 (1.4%)
Smoking status			
Current smoker	5 (6.8%)	9 (12.2%)	14 (9.5%)
Former smoker	39 (52.7%)	32 (43.2%)	71 (48.0%)
Never smoked	29 (39.2%)	33 (44.6%)	62 (41.9%)
Missing	1 (1.4%)	-	1 (0.7%)
Allocation			
Swab sampling	37 (50.0%)	37 (50.0%)	74 (50.0%)
Tissue sampling	37 (50.0%)	37 (50.0%)	74 (50.0%)
Diabetes Type			
Type 1	10 (13.5%)	7 (9.5%)	17 (11.5%)
Type 2	63 (85.1%)	67 (90.5%)	130 (87.8%)
Other—Monogenic	1 (1.4%)	-	1 (0.7%)
Duration of diabetes (years)			
Median (Interquartile Range)	15.5 (10.0 to 21.0)	15.5 (10.0 to 22.0)	15.5 (10.0 to 21.5)
Currently taking treatment for their diabetes? (not mutually-exclusive)			
Yes	74 (100.0%)	74 (100.0%)	148 (100.0%)
– Oral hypoglycaemic agent	46 (62.2%)	50 (67.6%)	96 (64.9%)
– Insulin	43 (58.1%)	40 (54.1%)	83 (56.1%)
– Other non-insulin injectables	6 (8.1%)	5 (6.8%)	11 (7.4%)
– Diet alone	7 (9.5%)	4 (5.4%)	11 (7.4%)
One DFU, or multiple			
One ulcer	57 (77.0%)	50 (67.6%)	107 (72.3%)
More than one ulcer	17 (23.0%)	24 (32.4%)	41 (27.7%)
Current pain score before sampling			
No pain	43 (58.1%)	41 (55.4%)	84 (56.8%)
Mild pain	20 (27.0%)	16 (21.6%)	36 (24.3%)
Moderate pain	7 (9.5%)	12 (16.2%)	19 (12.8%)
Severe pain	4 (5.4%)	5 (6.8%)	9 (6.1%)
Euroqol EQ5D-3L Responses at baseline			
Mobility			
No problems	22 (29.7%)	10 (13.5%)	32 (21.6%)
Some problems	47 (63.5%)	60 (81.1%)	107 (72.3%)
I am confined to bed	3 (4.1%)	2 (2.7%)	5 (3.4%)
No problems and Some problems selected	1 (1.4%)	1 (1.4%)	2 (1.4%)
Missing	1 (1.4%)	1 (1.4%)	2 (1.4%)
Self-care			
No problems	46 (62.2%)	42 (56.8%)	88 (59.5%)
Some problems	26 (35.1%)	26 (35.1%)	52 (35.1%)
Unable to wash or dress myself	1 (1.4%)	4 (5.4%)	5 (3.4%)
No problems and some problems selected	-	1 (1.4%)	1 (0.7%)
Missing	1 (1.4%)	1 (1.4%)	2 (1.4%)
Usual activities			
No problems	27 (36.5%)	24 (32.4%)	51 (34.5%)
Some problems	31 (41.9%)	37 (50.0%)	68 (45.9%)
Unable to perform my usual activities	14 (18.9%)	12 (16.2%)	26 (17.6%)
Missing	2 (2.7%)	1 (1.4%)	3 (2.0%)
Pain/discomfort			
I have no pain or discomfort	23 (31.1%)	19 (25.7%)	42 (28.4%)
I have moderate pain or discomfort	43 (58.1%)	40 (54.1%)	83 (56.1%)
I have extreme pain or discomfort	7 (9.5%)	14 (18.9%)	21 (14.2%)
Missing	1 (1.4%)	1 (1.4%)	2 (1.4%)
Anxiety/depression			
Not anxious or depressed	45 (60.8%)	37 (50.0%)	82 (55.4%)
Moderately anxious or depressed	27 (36.5%)	29 (39.2%)	56 (37.8%)
Extremely anxious or depressed	-	6 (8.1%)	6 (4.1%)
Missing	2 (2.7%)	2 (2.7%)	4 (2.7%)

Supplementary Appendix 4 summarises the baseline characteristics of those who were issued questionnaires at each of the timepoints in the two arms. The results are similar, although, it should be noted that participants who were issued the week 104 questionnaire will be from among those recruited at the beginning of the CODIFI2’s recruitment phase, and so this cohort may be systematically different to those recruited later due to unseen temporal trends, such as changes in recruitment practices (potentially related to COVID19) due to local site policies and or staff preferences.

### Outcomes

The questionnaire response rates are provided together in Table 2.

**Table 2:** Summary of questionnaire responses at weeks 39, 52 and 104

**Was the questionnaire returned**	**Standard letter**		**Enhanced letter**		
	**Received/sent**	**Response** **(%, 95% CI)**	**Received/sent**	**Response** **(%, 95% CI)**	**Difference in proportions (95% CI)**
Week 39	39/67	58.2% (45.5% to 70.2%)	47 / 67	70.1% (57.7% to 80.7%)	11.9% (− 5.7% to 29.1%)
Week 52	35/63	55.6% (42.5% to 68.1%)	45 / 64	70.3% (57.6% to 81.1%)	14.8% (− 3.2% to 31.2%)
Week 104	19/33	57.6% (39.2% to 74.5%)	24 / 33	72.7% (54.5% to 86.7%)	15.2% (− 10.4% to 39.3%)

At the 39-week follow-up, 67/74 (90.5%) participants in both arms were analysed due to having been sent a questionnaire pack. Reasons for questionnaires not being sent for this and all timepoints are included in the flow diagram (Fig. 1). The response rates were 47/67 (70.1%) and 39/67 (58.2%) for the enhanced and standard letter groups, respectively. The estimated difference in proportions responding was 11.9% (95% CI − 5.7% to 29.1%).

At the 52-week follow-up, 64/74 (86.5%) and 63/74 (85.1%) participants were sent a questionnaire in the enhanced and standard letter groups, respectively. Two participants at week 52 were removed from the week 52 analysis due to other reasons for not issuing a questionnaire (Fig. 1). The response rates were 45/64 (70.3%) vs 35/63 (55.6%) for the enhanced vs standard letters, respectively. The estimated difference in proportions responding was 14.8% (95% CI − 3.2% to 31.2%).

At the 104-week follow-up, 33/74 (44.6%) participants in each group were sent a questionnaire in the enhanced and standard letter groups, respectively. The response rates were 24/33 (72.7%) 19/33 (57.6%) for the enhanced vs standard letters, respectively. The estimated difference in proportions responding was 15.2% (95% CI − 10.4% to 39.3%).

### Updated *meta*-analysis

**Fig. 2:**
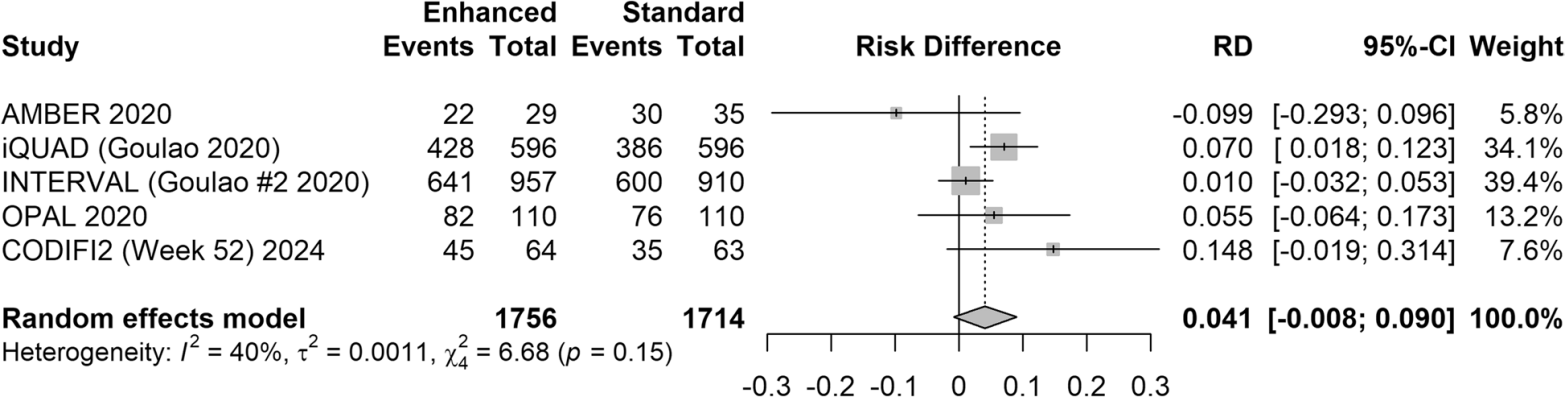
Forest plot of updated meta-analysis of randomised controlled trials of theoretically informed (Enhanced) cover letters embedded within randomised controlled trials. Footnote: positive risk differences (RD) indicate increase proportions responding when assigned to the ‘Enhanced’ (theoretically-informed) cover letter compared to the Standard letter. 95% confidence intervals for risk difference calculated using asymptotic method to align with Cochrane Review [10]. Unpublished AMBER (Abdominal massage for bowel dysfunction in people with multiple sclerosis) and OPAL (Optimal Pelvic floor muscle training for Adherence Long-term) SWAT results reported from ‘personal communications’ to Cochrane Review authors. CODIFI2 data taken from the week 52 timepoint. INTERVAL investigation of NICE technologies for enabling risk variable-adjusted length

In the 2021 Cochrane Review [10], four SWATs of a theoretically informed cover letter to improve response to mailed follow-up were included, including two unpublished findings provided by personal communications (Analysis 43 in [10]). The random effects meta-analysis of differences in response rates estimated a pooled difference in response rates (enhanced letter–standard letter) of 0.033 (95% CI − 0.015 to 0.080). Incorporating our 52-week results in this analysis (assuming that no other SWATs have been completed at time of publication), the updated pooled random effects estimate of difference in response rates (enhanced letter–standard letter) was 0.042 (95% CI − 0.009 to 0.092) (Tau-squared = 0.0012, *I*^2^ = 40.0%) (Fig. 2).

### Costs

We did not collect data prospectively on costs relating to this SWAT. However, from the intervention aspect, we note that the incremental cost of an enhanced letter will have been negligible, with no additional postage costs. The time spent on developing the enhanced letters’ content was trivial, as it was largely based on that from a previously existing SWAT, with changes suggested by PPI representatives.

Considering the costs of the SWAT, we did note that the SWAT incurred additional time to draft, implement and test the database report specifications that would generate the cover letters. A total of 15 different cover letter database reports were required covering combinations of timepoints, randomised allocation, past response and expected follow-up duration. A 16th, overarching administration report handled letter generation, as well as identifying those participants for whom a status check was required. Each database report incurred a time cost in terms of specification, implementation and testing the report. These costs occurred solely during study set up. Staff involved in generating the cover letters noted some increased challenges occurred with generating the letters. Specifically, the reporting system devised to generate the corresponding letters was not felt to be user-friendly, was felt to be ‘fiddly’ and ‘annoying’—requiring each letter to be generated one at a time on request by the user. Staff feedback indicated that this did not add significantly more work and did not allow generation of letters from the incorrect allocation group. Unfortunately, it was not possible to amend the system to fix these usability issues, so the system remained in place.

The only other cost to be considered was the need to perform additional ongoing monitoring and validation of the randomisation system for CODIFI2, owing to the need to monitor a second dynamic allocation system. This latter cost was much reduced, as standardised programmes to automate the checking of minimisation algorithms were already in place.

### Harms

No adverse events associated with the cover letter sub-study were reported.

## Discussion

In this embedded RCT of an enhanced, theoretically informed cover letter, we found that the enhanced cover letter appeared to increase the proportions of contacted participants responding at all three timepoints compared to the standard letter. However, the 95% confidence intervals for all estimated differences were wide and included the null value of no difference at all timepoints. No harms arising from the use of either cover letter were reported. The incremental costs of developing and implementing the enhanced letter were trivial, being based on a pre-existing template, and not requiring additional postage costs, respectively. The burden of running the SWAT did involve a fixed cost in terms of setting up the system to automatically generate letters, and our systems experienced usability issues, but there was not any considerable burden generated in terms of running the SWAT on a day-to-day basis. Our estimated differences in response rates were rather different to those in previously published SWATs [10, 11]. That said, our wide confidence intervals easily enclose the point estimates from the three largest prior studies. Further, the inclusion of our results did not materially increase the statistical measures of heterogeneity of the combined results.

### Strengths

The key strengths of this SWAT are that the groups were assigned at random, concealed allocation by means of a central independently operated randomisation service which was successful in ensuring balance in important demographic characteristics. Blinding was ensured both by withholding the assigned intervention from participants and site staff involved in all aspects of delivering the trial and delayed provision of the assigned intervention. We were also able to use our automated systems to efficiently generate the required cover letters.

### Limitations

The early termination of the CODIFI2 RCT (and, consequently, this SWAT) meant that the anticipated recruitment was lower than hoped for and so is a limitation of this SWAT. The result being that our estimated differences in response rates had low precision. Despite an apparently high observed difference in response rates, confidence intervals for this difference in response rates were wide. By itself, this ought not be a concern. SWATs are necessarily undertaken as embedded RCTs within (and secondary to) a host trial, with a view to being able to meta-analyse results, rather than as definitive standalone trials. These concerns also motivate reduced, simplified analysis plans, so as not to take time away from delivering the host RCT.

A possible limitation is that we randomised all participants to receive all letters of one kind at each follow-up and randomised all participants at the point of starting the host RCT, long before knowing if they would remain in follow up to begin receiving questionnaires. We chose this approach to minimise the impact on delivery of the host RCT. As a result of our design choice and consequent analysis of only those who were sent letters (rather than all randomised participants), our estimands are not those of a policy of enhanced covering letters, even in participants who are not followed up. Rather, we have estimated the effect of an enhanced letter in those who were sent a questionnaire pack. This is a departure from the ITT principle of including all participants in their randomised groups and so has the potential to introduce bias into our results [20].

An alternative approach might have been to re-randomise all active participants to a new letter at each available follow-up time [21]. In our case, this would have introduced extra challenges by increased delay in generating each follow-up letter. While the actual number recruited to CODIFI2 was small, had the host RCT recruited to target, the extra time needed to manually perform a randomisation for each letter that was due might have been substantial. In addition, from a design perspective, we were concerned that there may have been a cross-over or contamination effect: a participant receiving the more encouraging theory informed letter might have responded negatively to receiving the less encouraging standard letter at a later timepoint. These factors motivated our choice of a single randomisation to the same style of letter at all timepoints.

### Reflections and future research

Reflecting on our implementation of this SWAT in CODIFI2, we note that implementing the SWAT at several follow-up timepoints increased the complexity of the embedded RCT. An argument might be made for running this SWAT only at a single timepoint to minimise the set-up and running costs for the trial going forward. As a point of comparison, we consider again the 2021 Cochrane Review [10]. Of the four included SWATs using this intervention, the study characteristics for two studies (AMBER and INTERVAL [10, 11]) are clear that only a single follow-up timepoint formed part of the SWAT design. For the other two trials (iQUAD and OPAL [10, 11]), two different timepoints are mentioned. For all four trials, a single estimated effect is reported, rather than one for each timepoint where the SWAT was implemented. That said, our SWAT appears to be the only one so far that includes an evaluation of the effect at multiple distinct timepoints.

In light of Trial Forge Guidance [22] that further SWATs are needed until (at the very least) the GRADE assessment for all outcomes is ‘high’, it is clear that further SWATs testing this intervention are indicated. First, the 2021 Cochrane Review’s conclusion of ‘very low’ certainty of evidence for the benefit of this intervention [10] are unlikely to be changed to ‘high’ by these results, on account of the small sample size. Second, evaluation of the benefit of this intervention at multiple timepoints would be indicated, in order to determine if the effect of the enhanced cover letter is sustained.

Our decision to base all reminder letters on the Standard group’s mailing was in order to determine the effect of the initial mailing being changed to a theoretically informed design. Further studies could include the reminder letter in the package of letters to be re-designed and assigned at random. However, the four previous SWATs have not published their reminder letters, so we are unable to comment on the difference between our reminders and those used previously. It is also of interest that most participants opted in to receiving electronic reminders, though we were not able to explore whether this had any impact on response rates (or the effect of the assigned cover letter) due to constraints on the conduct and analysis of the RCT and SWAT.

We also consider that while our statistical analysis is in line with previously published work and the view that SWAT analyses need not be complex [23], further work might explore a number of potentially relevant objectives. For example, we are unable to perform subgroup analyses due to small sample sizes. We were time-constrained in delivering analysis to prevent us from exploring risk factors for non-return which may inform future study design. We did not consider whether increased return came at the cost of poorer questionnaire completion, which would still result in missing outcome data all the same. Finally, we did not explore whether reminder letters had any effect on return rates—including use of electronic reminders—and if this in any way mediated or moderated the effect of the intervention. All of these objectives may prove fruitful avenues for a suitable future SWAT.

Finally, we consider the consequences of adding our findings to the 2021 Cochrane Review. While we see a slight improvement in the pooled estimated effect of enhanced letters, we note that there is notable heterogeneity of effects across studies to date. More and larger RCTs of this intervention would yield a more precise estimate of the mean effect of theory-informed cover letters and provide a clearer picture of the heterogeneity of effects in other populations. While we could have included all three timepoints in our update, we declined to do so since our results are not independent: a key assumption in synthesising research findings.

## Conclusion

Participants sent enhanced cover letters in CODIFI2 appeared to have a numerically higher response rate to questionnaires at all timepoints than those sent standard cover letters. However, the estimated effects are uncertain, due to small sample sizes. Despite this, these results of this SWAT seem compatible with other findings previously reported and synthesised. The effect of adding our results to the Gillies et al. Cochrane Review [10] has the effect of slightly improving the pooled estimated benefit of theory-informed letters. Taken together the findings from this SWAT, when included alongside other replications of SWATs of enhanced, theoretically informed cover letters are suggestive of benefit due to enhanced cover letters exploiting behavioural change theory to encourage response. However, further research in more and larger RCTs that include multiple analysis timepoints would be required to provide clearer evidence of benefit (or lack thereof) and so promote changes to conduct of RCTs.

## Supplementary Information

Additional file 1: Supplementary Appendix 1 - Eligibility Criteria for the CODIFI2 host trial and cover letter SWAT

Additional file 2: Supplementary Appendix 2 - Cover Letter Bundle

Additional file 3: Supplementary Appendix 3 - Email and SMS reminders

Additional file 4: Supplementary Appendix 4 - Baseline characteristics by SWAT randomised arm among those sent questionnaires at each time point

## Data Availability

Data supporting this work are available on reasonable request. All requests will be reviewed by relevant stakeholders, based on the principles of a controlled access approach. Requests to access data should be made to CTRU-DataAccess@leeds.ac.uk in the first instance.

## Abbreviations

AMBER: Abdominal massage for bowel dysfunction in people with multiple sclerosis (trial acronym)
CI: Confidence interval
CODIFI2: Concordance in diabetic foot infection 2 (trial acronym)
COVID19: Coronavirus disease 2019
CTRU: Clinical Trials Research Unit
DFU: Diabetic foot ulcer
GRADE: Grading of Recommendations, Assessment, Development, and Evaluations
INTERVAL: Investigation of NICE technologies for enabling risk-variable-adjusted-length (trial acronym)
iQUAD: Improving the Quality of Dentistry (trial acronym)
ISRCTN: International Standard Randomised Controlled Trial Number
LADDER: Lay ADvice on Diabetes and Endocrine Research
NHS: National Health Service
NIHR: National Institute for Healthcare Research
OPAL: Optimal Pelvic floor muscle training for Adherence Long-term (trial acronym)
PRioRiTy II: Prioritising Retention in Randomised Trials
PPI: Participant and public involvement
RCT: Randomised controlled trial
RD: Risk difference
REC: Research Ethics Committee
SMS: Short message service
SWAT: Study within a trial
UK: United Kingdom
