# Supplementary material for: Theoretically-informed vs standard cover letter to improve participant response to mailed questionnaire: results of an embedded randomised retention trial
Source: Trials. 2024 Nov 14;25:763. doi: 10.1186/s13063-024-08565-0 (PMC11562673; doi:10.1186/s13063-024-08565-0)
Supplement: Supplementary file 3 — Additional file 3: Supplementary Appendix 3 - Email and SMS reminders [file 13063_2024_8565_MOESM3_ESM.docx]

Supplementary Material, accompanying

Theoretically-informed vs standard cover letter to improve participant response to mailed questionnaire: results of an embedded randomised retention trial

Colin C Everett, Sarah T Brown, Joanna L Dennett, Howard Collier, Claire L Davies, Frances Game, E Andrea Nelson.

**Supplementary Appendix 3: SMS and Email reminders sent to participants**

## Introduction

For those participants who opted in to electronic reminders, the CODIFI2 trial issued SMS and / or email reminders. The contents of these reminders (with phone numbers and trial email addresses redacted from this supplement) were as follows:

## Email Reminder Message

Sender Email: XXXXXXXXXX@leeds.ac.uk

Subject: CODIFI 2 Quality of Life questionnaires

Dear Participant

You have kindly agreed to take part in the CODIFI 2 study. This is a very important study looking at sampling methods for infected diabetic foot ulcers and the effect a diabetic foot ulcer has on people's quality of life.

We recently sent you a questionnaire pack to complete but unfortunately we have not received the completed questionnaires back as yet. We would be very grateful if you could complete and return them as soon as possible.

To return the questionnaires please use the stamped address envelope that was enclosed in the pack.

If you have changed address recently or have not received the questionnaire pack, please contact us on XXXX XXX XXXX or email XXXXXXXXXX@leeds.ac.uk

Thank you for your help with this important study, it is greatly appreciated.

Yours Sincerely

CODIFI 2 research team

## SMS Reminder Message

We recently sent you a questionnaire, if you have not received it please contact us on XXXX XXX XXXX. Your questionnaire responses are important to us.
