# Supplementary material for: Theoretically-informed vs standard cover letter to improve participant response to mailed questionnaire: results of an embedded randomised retention trial
Source: Trials. 2024 Nov 14;25:763. doi: 10.1186/s13063-024-08565-0 (PMC11562673; doi:10.1186/s13063-024-08565-0)
Supplement: Supplementary file 4 — Additional file 4: Supplementary Appendix 4 - Baseline characteristics by SWAT randomised arm among those sent questionnaires at each time point [file 13063_2024_8565_MOESM4_ESM.docx]

Supplementary Material, accompanying

Theoretically-informed vs standard cover letter to improve participant response to mailed questionnaire: results of an embedded randomised retention trial

Colin C Everett, Sarah T Brown, Joanna L Dennett, Howard Collier, Claire L Davies, Frances Game, E Andrea Nelson.

**Supplementary Appendix 4: Baseline Characteristics by arm for those still on study at each timepoint**

## Introduction

The table of baseline characteristics in the main body summarises baseline characteristics in all randomised participants. Since the analysis excludes participants not sent letters at each timepoint, the following tables provides the baseline characteristics by arm for each of the analysis sets.

|  | **Sent a letter at week 39** | | | **Sent a letter at week 52** | | | **Sent a letter at week 104** | | |
| --- | --- | --- | --- | --- | --- | --- | --- | --- | --- |
| **Variable** | **Standard Letter (n=67)** | **Enhanced Letter (n=67)** | **Total (n=134)** | **Standard Letter (n=63)** | **Enhanced Letter (n=64)** | **Total (n=127)** | **Standard Letter (n=33)** | **Enhanced Letter (n=33)** | **Total (n=66)** |
| **Age, years** |  |  |  |  |  |  |  |  |  |
| Mean (SD) | 61.3 (11.78) | 62.4 (11.70) | 61.9 (11.71) | 61.3 (11.80) | 62.2 (11.39) | 61.8 (11.56) | 60.0 (12.08) | 59.9 (10.00) | 59.9 (11.00) |
| Range | (31 to 83.0) | (32 to 92) | (31 to 92) | (31 to 83) | (32 to 92) | (31 to 92) | (31 to 81) | (32 to 75) | (31 to 81) |
|  |  |  |  |  |  |  |  |  |  |
| **Gender** |  |  |  |  |  |  |  |  |  |
| Male | 56 (83.6%) | 56 (83.6%) | 112 (83.6%) | 52 (82.5%) | 53 (82.8%) | 105 (82.7%) | 26 (78.8%) | 29 (87.9%) | 55 (83.3%) |
| Female | 11 (16.4%) | 11 (16.4%) | 22 (16.4%) | 11 (17.5%) | 11 (17.2%) | 22 (17.3%) | 7 (21.2%) | 4 (12.1%) | 11 (16.7%) |
|  |  |  |  |  |  |  |  |  |  |
| **Ethnicity** |  |  |  |  |  |  |  |  |  |
| White | 64 (95.5%) | 65 (97.0%) | 129 (96.3%) | 60 (95.2%) | 62 (96.9%) | 122 (96.1%) | 33 (100.0%) | 32 (97.0%) | 65 (98.5%) |
| Mixed - White and Black Caribbean | - | 1 (1.5%) | 1 (0.7%) | - | 1 (1.6%) | 1 (0.8%) | - | 1 (3.0%) | 1 (1.5%) |
| Asian - Pakistani | - | 1 (1.5%) | 1 (0.7%) | - | 1 (1.6%) | 1 (0.8%) | - | - | - |
| Other Asian background | 1 (1.5%) | - | 1 (0.7%) | 1 (1.6%) | - | 1 (0.8%) | - | - | - |
| Black - African | 2 (3.0%) | - | 2 (1.5%) | 2 (3.2%) | - | 2 (1.6%) | - | - | - |
|  |  |  |  |  |  |  |  |  |  |
| **Smoking Status** |  |  |  |  |  |  |  |  |  |
| Current smoker | 5 (7.5%) | 7 (10.4%) | 12 (9.0%) | 5 (7.9%) | 7 (10.9%) | 12 (9.4%) | 2 (6.1%) | 4 (12.1%) | 6 (9.1%) |
| Former smoker | 35 (52.2%) | 29 (43.3%) | 64 (47.8%) | 34 (54.0%) | 29 (45.3%) | 63 (49.6%) | 16 (48.5%) | 13 (39.4%) | 29 (43.9%) |
| Never smoked | 27 (40.3%) | 31 (46.3%) | 58 (43.3%) | 24 (38.1%) | 28 (43.8%) | 52 (40.9%) | 15 (45.5%) | 16 (48.5%) | 31 (47.0%) |
|  |  |  |  |  |  |  |  |  |  |
| **Allocation** |  |  |  |  |  |  |  |  |  |
| Swab sampling | 31 (46.3%) | 34 (50.7%) | 65 (48.5%) | 30 (47.6%) | 32 (50.0%) | 62 (48.8%) | 15 (45.5%) | 15 (45.5%) | 30 (45.5%) |
| Tissue sampling | 36 (53.7%) | 33 (49.3%) | 69 (51.5%) | 33 (52.4%) | 32 (50.0%) | 65 (51.2%) | 18 (54.5%) | 18 (54.5%) | 36 (54.5%) |
|  |  |  |  |  |  |  |  |  |  |
| **Diabetes Type** |  |  |  |  |  |  |  |  |  |
| Type 1 | 10 (14.9%) | 6 (9.0%) | 16 (11.9%) | 10 (15.9%) | 5 (7.8%) | 15 (11.8%) | 7 (21.2%) | 4 (12.1%) | 11 (16.7%) |
| Type 2 | 56 (83.6%) | 61 (91.0%) | 117 (87.3%) | 52 (82.5%) | 59 (92.2%) | 111 (87.4%) | 26 (78.8%) | 29 (87.9%) | 55 (83.3%) |
| Other - Monogenic | 1 (1.5%) | - | 1 (0.7%) | 1 (1.6%) | - | 1 (0.8%) | - | - | - |
|  |  |  |  |  |  |  |  |  |  |
| **Duration of diabetes (years)** |  |  |  |  |  |  |  |  |  |
| Median (Interquartile Range) | 15.0   (10.0 to 24.0) | 15.0   (10.0 to 20.0) | 15.0   (10.0 to 21.0) | 15.0   (10.0 to 24.0) | 15.0   (10.5 to 20.0) | 15.0   (10.0 to 21.0) | 13.0   (8.0 to 20.0) | 15.0   (13.0 to 20.0) | 15.0   (10.0 to 20.0) |
|  |  |  |  |  |  |  |  |  |  |
| **Currently taking treatment for their diabetes? (not mutually-exclusive)** |  |  |  |  |  |  |  |  |  |
| Yes | 67 (100.0%) | 67 (100.0%) | 134 (100.0%) | 63 (100.0%) | 64 (100.0%) | 127 (100.0%) | 33 (100.0%) | 33 (100.0%) | 66 (100.0%) |
| -- Oral hypoglycaemic agent | 42 (62.7%) | 46 (68.7%) | 88 (65.7%) | 40 (63.5%) | 44 (68.8%) | 84 (66.1%) | 22 (66.7%) | 21 (63.6%) | 43 (65.2%) |
| -- Insulin | 39 (58.2%) | 36 (53.7%) | 75 (56.0%) | 37 (58.7%) | 34 (53.1%) | 71 (55.9%) | 17 (51.5%) | 17 (51.5%) | 34 (51.5%) |
| -- Other non-insulin injectables | 6 (9.0%) | 5 (7.5%) | 11 (8.2%) | 6 (9.5%) | 5 (7.8%) | 11 (8.7%) | 3 (9.1%) | 3 (9.1%) | 6 (9.1%) |
| -- Diet alone | 5 (7.5%) | 4 (6.0%) | 9 (6.7%) | 4 (6.3%) | 4 (6.3%) | 8 (6.3%) | 2 (6.1%) | 3 (9.1%) | 5 (7.6%) |
|  |  |  |  |  |  |  |  |  |  |
| **One DFU, or multiple** |  |  |  |  |  |  |  |  |  |
| One ulcer | 52 (77.6%) | 44 (65.7%) | 96 (71.6%) | 50 (79.4%) | 42 (65.6%) | 92 (72.4%) | 25 (75.8%) | 22 (66.7%) | 47 (71.2%) |
| More than one ulcer | 15 (22.4%) | 23 (34.3%) | 38 (28.4%) | 13 (20.6%) | 22 (34.4%) | 35 (27.6%) | 8 (24.2%) | 11 (33.3%) | 19 (28.8%) |
|  |  |  |  |  |  |  |  |  |  |
| **Current pain score before sampling** |  |  |  |  |  |  |  |  |  |
| No Pain | 41 (61.2%) | 35 (52.2%) | 76 (56.7%) | 38 (60.3%) | 33 (51.6%) | 71 (55.9%) | 17 (51.5%) | 18 (54.5%) | 35 (53.0%) |
| Mild Pain | 15 (22.4%) | 15 (22.4%) | 30 (22.4%) | 14 (22.2%) | 14 (21.9%) | 28 (22.0%) | 10 (30.3%) | 5 (15.2%) | 15 (22.7%) |
| Moderate Pain | 7 (10.4%) | 12 (17.9%) | 19 (14.2%) | 7 (11.1%) | 12 (18.8%) | 19 (15.0%) | 5 (15.2%) | 7 (21.2%) | 12 (18.2%) |
| Severe Pain | 4 (6.0%) | 5 (7.5%) | 9 (6.7%) | 4 (6.3%) | 5 (7.8%) | 9 (7.1%) | 1 (3.0%) | 3 (9.1%) | 4 (6.1%) |
|  |  |  |  |  |  |  |  |  |  |
| **Mobility** |  |  |  |  |  |  |  |  |  |
| No problems | 21 (31.3%) | 9 (13.4%) | 30 (22.4%) | 21 (33.3%) | 9 (14.1%) | 30 (23.6%) | 12 (36.4%) | 2 (6.1%) | 14 (21.2%) |
| Some problems | 42 (62.7%) | 54 (80.6%) | 96 (71.6%) | 38 (60.3%) | 51 (79.7%) | 89 (70.1%) | 19 (57.6%) | 29 (87.9%) | 48 (72.7%) |
| I am confined to bed | 2 (3.0%) | 2 (3.0%) | 4 (3.0%) | 2 (3.2%) | 2 (3.1%) | 4 (3.1%) | 1 (3.0%) | 1 (3.0%) | 2 (3.0%) |
| No problems and Some problems selected | 1 (1.5%) | 1 (1.5%) | 2 (1.5%) | 1 (1.6%) | 1 (1.6%) | 2 (1.6%) | - | - | - |
| Missing | 1 (1.5%) | 1 (1.5%) | 2 (1.5%) | 1 (1.6%) | 1 (1.6%) | 2 (1.6%) | 1 (3.0%) | 1 (3.0%) | 2 (3.0%) |
|  |  |  |  |  |  |  |  |  |  |
| **Self-care** |  |  |  |  |  |  |  |  |  |
| No problems | 43 (64.2%) | 39 (58.2%) | 82 (61.2%) | 42 (66.7%) | 36 (56.3%) | 78 (61.4%) | 23 (69.7%) | 19 (57.6%) | 42 (63.6%) |
| Some problems | 22 (32.8%) | 22 (32.8%) | 44 (32.8%) | 19 (30.2%) | 22 (34.4%) | 41 (32.3%) | 8 (24.2%) | 12 (36.4%) | 20 (30.3%) |
| Unable to wash or dress myself | 1 (1.5%) | 4 (6.0%) | 5 (3.7%) | 1 (1.6%) | 4 (6.3%) | 5 (3.9%) | 1 (3.0%) | - | 1 (1.5%) |
| No problems and Some problems selected | - | 1 (1.5%) | 1 (0.7%) | - | 1 (1.6%) | 1 (0.8%) | - | 1 (3.0%) | 1 (1.5%) |
| Missing | 1 (1.5%) | 1 (1.5%) | 2 (1.5%) | 1 (1.6%) | 1 (1.6%) | 2 (1.6%) | 1 (3.0%) | 1 (3.0%) | 2 (3.0%) |
|  |  |  |  |  |  |  |  |  |  |
| **Usual Activities** |  |  |  |  |  |  |  |  |  |
| No problems | 26 (38.8%) | 23 (34.3%) | 49 (36.6%) | 26 (41.3%) | 23 (35.9%) | 49 (38.6%) | 12 (36.4%) | 7 (21.2%) | 19 (28.8%) |
| Some problems | 28 (41.8%) | 32 (47.8%) | 60 (44.8%) | 26 (41.3%) | 29 (45.3%) | 55 (43.3%) | 13 (39.4%) | 20 (60.6%) | 33 (50.0%) |
| Unable to perform my usual activities | 11 (16.4%) | 11 (16.4%) | 22 (16.4%) | 9 (14.3%) | 11 (17.2%) | 20 (15.7%) | 6 (18.2%) | 5 (15.2%) | 11 (16.7%) |
| Missing | 2 (3.0%) | 1 (1.5%) | 3 (2.2%) | 2 (3.2%) | 1 (1.6%) | 3 (2.4%) | 2 (6.1%) | 1 (3.0%) | 3 (4.5%) |
|  |  |  |  |  |  |  |  |  |  |
| **Pain / Discomfort** |  |  |  |  |  |  |  |  |  |
| No pain or discomfort | 23 (34.3%) | 18 (26.9%) | 41 (30.6%) | 23 (36.5%) | 18 (28.1%) | 41 (32.3%) | 10 (30.3%) | 8 (24.2%) | 18 (27.3%) |
| Moderate pain or discomfort | 38 (56.7%) | 36 (53.7%) | 74 (55.2%) | 35 (55.6%) | 33 (51.6%) | 68 (53.5%) | 20 (60.6%) | 17 (51.5%) | 37 (56.1%) |
| Extreme pain or discomfort | 5 (7.5%) | 12 (17.9%) | 17 (12.7%) | 4 (6.3%) | 12 (18.8%) | 16 (12.6%) | 2 (6.1%) | 7 (21.2%) | 9 (13.6%) |
| Missing | 1 (1.5%) | 1 (1.5%) | 2 (1.5%) | 1 (1.6%) | 1 (1.6%) | 2 (1.6%) | 1 (3.0%) | 1 (3.0%) | 2 (3.0%) |
|  |  |  |  |  |  |  |  |  |  |
| **Anxiety / Depression** |  |  |  |  |  |  |  |  |  |
| Not anxious or depressed | 42 (62.7%) | 33 (49.3%) | 75 (56.0%) | 42 (66.7%) | 30 (46.9%) | 72 (56.7%) | 22 (66.7%) | 18 (54.5%) | 40 (60.6%) |
| Moderately anxious or depressed | 23 (34.3%) | 28 (41.8%) | 51 (38.1%) | 19 (30.2%) | 28 (43.8%) | 47 (37.0%) | 10 (30.3%) | 13 (39.4%) | 23 (34.8%) |
| Extremely anxious or depressed | - | 4 (6.0%) | 4 (3.0%) | - | 4 (6.3%) | 4 (3.1%) | - | 1 (3.0%) | 1 (1.5%) |
| Missing | 2 (3.0%) | 2 (3.0%) | 4 (3.0%) | 2 (3.2%) | 2 (3.1%) | 4 (3.1%) | 1 (3.0%) | 1 (3.0%) | 2 (3.0%) |
|  |  |  |  |  |  |  |  |  |  |
