# Supplementary material for: Theoretically-informed vs standard cover letter to improve participant response to mailed questionnaire: results of an embedded randomised retention trial
Source: Trials. 2024 Nov 14;25:763. doi: 10.1186/s13063-024-08565-0 (PMC11562673; doi:10.1186/s13063-024-08565-0)
Supplement: Supplementary file 1 — Additional file 1: Supplementary Appendix 1 - Eligibility Criteria for the CODIFI2 host trial and cover letter SWAT [file 13063_2024_8565_MOESM1_ESM.pdf]

Supplementary Material, accompanying

Theoretically-informed vs standard cover letter to improve participant response to mailed questionnaire: results of an embedded randomised retention trial

Colin C Everett, Sarah T Brown, Joanna L Dennett, Howard Collier, Claire L Davies, Frances Game, E Andrea Nelson.

Supplementary Appendix 1: Eligibility Criteria

**Inclusion Criteria**	**Exclusion Criteria**
18 years of age or older at the time of signing the consent form;<br>Diagnosis of diabetes mellitus (according to WHO criteria [a]);<br>Presence of a DFU with a suspected mild to moderate soft tissue infection (as per IDSA guidelines [b]);<br>Able and willing to provide informed consent for participation in the study;<br>Consent for foot photography.	Index ulcer present for >2 years;<br>Presence of suspected osteomyelitis of the index limb;<br>Previous participation in the trial;<br>Not expected to comply with the sampling strategies (i.e., has a preference);<br>Not expected to comply with the follow-up schedule;<br>In the opinion of the local investigator the participant's foot infection is too severe to include them in the study.

Note, no additional criteria were required to be randomised to the SWAT.

References:

a. World Health Organization. (2006) Definition and diagnosis of diabetes mellitus and intermediate hyperglycaemia: report of a WHO/IDF consultation. WHO Document Production Services, Geneva, Switzerland. ISBN: 9241594934 https://www.who.int/publications/i/item/definition-and-diagnosis-of-diabetes-mellitus-and-intermediate-hyperglycaemia (Accessed 2nd February 2024)

b. Lipsky BA, Berendt AR, Cornia PB et al. 2012 Infectious Diseases Society of America Clinical Practice Guideline for the Diagnosis and Treatment of Diabetic Foot Infections, Clinical Infectious Diseases, Volume 54, Issue 12, 15 June 2012, Pages e132–e173, https://doi.org/10.1093/cid/cis346
