# Supplementary material for: Theoretically-informed vs standard cover letter to improve participant response to mailed questionnaire: results of an embedded randomised retention trial
Source: Trials. 2024 Nov 14;25:763. doi: 10.1186/s13063-024-08565-0 (PMC11562673; doi:10.1186/s13063-024-08565-0)
Supplement: Supplementary file 2 — Additional file 2: Supplementary Appendix 2 - Cover Letter Bundle [file 13063_2024_8565_MOESM2_ESM.pdf]

Supplementary Material, accompanying

Theoretically-informed vs standard cover letter to improve participant response to mailed questionnaire: results of an embedded randomised retention trial

Colin C Everett, Sarah T Brown, Joanna L Dennett, Howard Collier, Claire L Davies, Frances Game, E Andrea Nelson.

Supplementary Appendix 2: Cover Letter package

Introduction

This document comprises templates of the cover letters issued as part of the CODIFI2 cover letter study within a trial (SWAT). This SWAT randomly assigned participants to receive covering letters confirming to a standard form, or to an enhanced form, with contents informed by behavioural change theory.

The following cover letters were generated to accompany questionnaire booklets at the following timepoints:

<table>
<tr><th>Timepoint</th><th>Standard</th><th>Enhanced</th></tr>
<tr><td>Week 39</td><td>W39Standard</td><td>W39Enhanced</td></tr>
<tr><td>Week 52</td><td>2 options, depending on expected followup duration:
1) If W104 visit expected: W52 Standard;
2) If W104 not expected: W52 StandardFinal.</td><td>4 options, depending on expected followup duration, and receipt of W39 pack:
1) If W39 pack not returned and W104 visit expected: W52Enhanced;
2) If W39 pack returned and W104 visit expected: W52EnhancedThanks;
3) If W39 pack not returned and W104 visit not expected: W52EnhancedFinal
4) If W39 pack returned and W104 visit not expected: W52EnhancedFinalThanks</td></tr>
<tr><td>Week 104</td><td>W104Standard</td><td>W104Enhanced</td></tr>
<tr><td>Reminder (non-response)</td><td colspan="2">Reminder</td></tr>
</table>

Notes

Letters are presented in chronological order, with Standard letters being followed by their corresponding Enhanced letters. The Reminder is the final letter in this bundle.

Specific cover letters are identified by watermarks at the top and right side of the following pages. These watermarks have been added to this bundle to aid identification, and were not present when issued to participants.

All letters except for the reminder letter included the same photograph of the same person. This photo has been replaced with an anonymised picture within this bundle.

The reminder letter was the only letter that included any logos for the University of Leeds and/or the Clinical Trials Research Unit. None of the other letters required University of Leeds (or CTRU) letterhead. There were three different database reports for reminder letters, 1 for each timepoint. However, since the three letters were identical, this is only included once in the pack.

All letters used Arial typeface, 11pt size, with single line spacing. Where possible, a letter was designed to fit to a single page. However, one of the W52 Enhanced letters required 2 pages.

We also prepared two further cover letters (standard and enhanced) to accompany booklets issued to participants who had withdrawn from clinical assessments and research visits, but were willing to receive mailed questionnaires. Ultimately, these were not issued to participants, and so are not included in this pack.

W39 Standard

dd-mm-yyyy

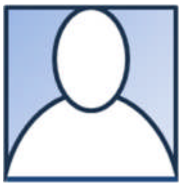

Mr/Miss/Ms Xxxxx Xxxxx
XXX Xxxxx Xxxxx
Xxxxx
XX XXX

Professor Andrea Nelson
Chief Investigator, CODIFI2

Dear Xxxx

Concordance in Diabetic Foot Ulcer Infection – 2 (CODIFI2)

I am writing to you to follow up on a research study that you very kindly agreed to take part in when you visited <Site Name> around 9 months ago. This study is called CODIFI2, and looks to see whether infected diabetic foot ulcers heal quicker if samples are taken by swabbing or taking tissue from the affected area.

Following your initial visit, you agreed to complete some follow-up questionnaires in the following months. Please take time to complete this questionnaire and return in the provided stamped-addressed envelope.

We would be grateful if you could also return the most recent copy of your Antibiotic Diary, even if it is blank.

If you have any queries about the questionnaire please don’t hesitate to contact Xxxx Xxxx at the University of Leeds on Xxxx@Leeds.ac.uk. Further information about the study can be found on our website https://ctru.leeds.ac.uk/codifi2

Thank you for your help with this study.

Yours sincerely,

Andrea Nelson
Chief Investigator, CODIFI2

W39 Standard

W39 Enhanced

dd-mm-yyyy

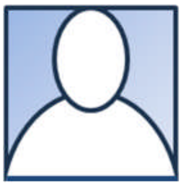

Professor Andrea Nelson
Chief Investigator, CODIFI2

Mr/Miss/Ms Xxxxx Xxxxx
XXX Xxxxx Xxxxx
Xxxxx
Xxxxx
XX XXX

Dear Mr/Miss/Ms Xxxxx Xxxxx

Concordance in Diabetic Foot Ulcer Infection – 2 (CODIFI2)

I am writing to you to follow up on a research study that you very kindly agreed to take part in following a visit to <Site Name> around 9 months ago to look at your diabetic foot ulcers. This study is called CODIFI2, and looks to see whether infected diabetic foot ulcers heal quicker if samples are taken by swabbing or by taking tissue from the affected area. This study is supported by the NHS' National Institute for Healthcare Research and the University of Leeds, and involves hospitals across Britain. If this trial is successful, the results may lead to changes in hospital practices to better manage the diagnosis of people like you, and may lead to better use of antibiotics.

We would like to know how you are feeling, 9 months after your initial visit. We are keen to hear from you, no matter how your health has changed in this time. To know this, we would like you to please complete the enclosed questionnaire booklet, and return it to us in the stamped addressed envelope provided. We would be very grateful if you could complete the questionnaire and return it to us as soon as possible. There are no right or wrong answers to these questions. Your answers will give a broad view of the impact of having a diabetic foot ulcer as time goes on. Neither your GP nor staff at the hospital you attended will be able to see your answers, so you do not need to worry about what they might think of your answers. If you have any questions, please feel free to get in touch with Xxxxx Xxxxx at the University of Leeds on Xxxxx@Leeds.ac.uk. Further information about the study can be found on our website https://ctru.leeds.ac.uk/codifi2

We would be grateful if you could also return the most recent copy of your Antibiotic Diary, even if it is blank.

We really appreciate your answers - no matter how you complete the questionnaire - as they give us the best idea of your quality of life after your first visit. Your responses are important, as we need them to make sure that the results are as reliable as possible and may help to change clinical practice for the better!

Thank you for your help with this study. We will contact you again with another questionnaire in 3 months time to ask how you are getting on.

Yours sincerely,

Andrea Nelson
Chief Investigator, CODIFI2

W39 Enhanced

W52 Standard

Mr/Miss/Ms Xxxx Xxxxx
XXX Xxxxx Xxxx
Xxxxxx
Xxxxxx
XXX XXX

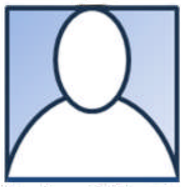

Professor Andrea Nelson
Chief Investigator, CODIFI2

dd-mm-yyyy

Dear Mr/Miss/Ms Xxxxx Xxxxx

Title: Concordance in Diabetic Foot Ulcer Infection – 2 (CODIFI2)

I am writing to you to follow up on a research study that you very kindly agreed to take part in when you visited <Site Name> around a year ago. This study is called CODIFI2, and looks to see whether infected diabetic foot ulcers heal quicker if samples are taken by swabbing or taking tissue from the affected area.

Following your initial visit, you agreed to complete some follow-up questionnaires in the following months. Please take time to complete this questionnaire and return in the provided stamped-addressed envelope.

We would be grateful if you could also return the most recent copy of your Antibiotic Diary, even if it is blank.

If you have any queries about the questionnaire please don't hesitate to contact Xxxxx Xxxxx at the University of Leeds on Xxxxx@Leeds.ac.uk. Further information about the study can be found on our website https://ctru.leeds.ac.uk/codifi2

Thank you for your help with this study.

Yours sincerely,

Andrea Nelson
Chief Investigator, CODIFI2

W52 Standard

W52 Standard Final

CODIFI2 Comparing sampling & processing methods in diabetic foot infections

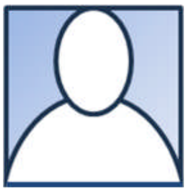

Mr/Miss/Ms Xxxx Xxxx
XXX Xxxx Xxxx
Xxxxxxx
Xxxxxxx
XXX XXX
dd-mm-yyyy

Professor Andrea Nelson
Chief Investigator, CODIFI2

Dear Mr/Miss/Ms Xxxxx Xxxxx

Concordance in Diabetic Foot Ulcer Infection – 2 (CODIFI2)

I am writing to you to follow up on a research study that you very kindly agreed to take part in when you visited <Site Name> around a year ago. This study is called CODIFI2, and looks to see whether infected diabetic foot ulcers heal quicker if samples are taken by swabbing or taking tissue from the affected area.

Following your initial visit, you agreed to complete some follow-up questionnaires in the following months. Please take time to complete this questionnaire and return in the provided stamped-addressed envelope. This is the last questionnaire for this study.

We would be grateful if you could also return the most recent copy of your Antibiotic Diary, even if it is blank.

If you have any queries about the questionnaire please don't hesitate to contact Xxxxx Xxxxx at the University of Leeds on Xxxxx@Leeds.ac.uk. Further information about the study can be found on our website https://ctru.leeds.ac.uk/codifi2

Thank you for your help with this study.

Yours sincerely,

Andrea Nelson
Chief Investigator, CODIFI2

W52 Standard Final

W52 Enhanced

Mr/Miss/Ms Xxxx Xxxxx
XXX Xxxx Xxxxx
Xxxxx
Xxxxx
XXX XXX

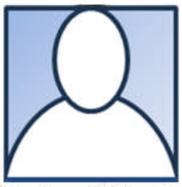

Professor Andrea Nelson
Chief Investigator, CODIFI2

W52 Enhanced

dd-mm-yyyy

Dear Mr/Miss/Ms Xxxx Xxxxx

Title: Concordance in Diabetic Foot Ulcer Infection – 2 (CODIFI2)

I am writing to you to follow up on the CODIFI2 research study that you very kindly agreed to take part in following a visit to <Site Name> around 12 months ago to look at your diabetic foot ulcers. CODIFI2 looks to see whether infected diabetic foot ulcers heal quicker if samples are taken by swabbing or by taking tissue from the affected area. This study is supported by the NHS' National Institute for Healthcare Research and the University of Leeds, and involves hospitals across Britain. If this trial is successful, the results may lead to changes in hospital practices to better manage the diagnosis of people like you, and may lead to better use of antibiotics.

It is now a year since you first agreed to take part in the study, and I am writing to provide the last questionnaire booklet for you to complete. We would now like to know how you are feeling, 12 months after your initial visit. We are keen to hear from you, no matter how your health has changed in this time. To know this, we would like you to complete the enclosed questionnaire, and return it to us in the envelope provided. We would be very grateful if you could complete the questionnaire and return it to us as soon as possible. There are no right or wrong answers to these questions. Your answers will give a broad view of the impact of having a diabetic foot ulcer as time goes on. Neither your GP nor staff at the hospital you attended will be able to see your answers, so you do not need to worry about what they might think of your answers. If you have any questions, please feel free to get in touch with Xxxx Xxxx at the University of Leeds on Xxxx@Leeds.ac.uk. Further information about the study can be found on our website https://ctru.leeds.ac.uk/codifi2/

We would be grateful if you could also return the most recent copy of your Antibiotic Diary, even if it is blank.

We really appreciate your answers - no matter how you complete the questionnaire - as they give us the best idea of your quality of life after your first visit. Your responses are important, as we need them to make sure that the results are as reliable as possible and may help to change clinical practice for the better!

W52 Enhanced

Thank you for help with this study. We will contact you again with the last questionnaire booklet in 12 months time to ask how you are getting on.

Yours sincerely

Angela Nelson
Chief Investigator, CODIFI2

W52 Enhanced

W52 Enhanced (with thanks)

dd-mm-yyyy

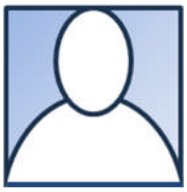

Mr/Miss/Ms Xxxx Xxxxx
XX Xxxx Xxxx
Xxxx
Xxxx
XXX XXX

Professor Andrea Nelson
Chief Investigator, CODIFI2

W52 Enhanced (with thanks)

Dear Xxxxx

Concordance in Diabetic Foot Ulcer Infection – 2 (CODIFI2)

I am writing to you to follow up on a research study that you very kindly agreed to take part in following a visit to <Site Name> around 12 months ago to look at your diabetic foot ulcers. CODIFI2 looks to see whether infected diabetic foot ulcers heal quicker if samples are taken by swabbing or by taking tissue from the affected area. This study is supported by the NHS' National Institute for Healthcare Research and the University of Leeds, and involves hospitals across Britain. If this trial is successful, the results may lead to changes in hospital practices to better manage the diagnosis of people like you, and may lead to better use of antibiotics.

Thank you for completing and returning your 9 month questionnaire booklet. We would now like to know how you are feeling, 12 months after your initial visit. We are keen to hear from you, no matter how your health has changed in this time. To know this, we would like you to complete the enclosed questionnaire, and return it to us in the envelope provided. We would be very grateful if you could complete the questionnaire and return it to us as soon as possible. There are no right or wrong answers to these questions. Your answers will give a broad view of the impact of having a diabetic foot ulcer as time goes on. Neither your GP nor staff at the hospital you attended will be able to see your answers, so you do not need to worry about what they might think of your answers. If you have any questions, please feel free to get in touch with Xxxx Xxxx at the University of Leeds on Xxxx@Leeds.ac.uk. Further information about the study can be found on our website https://ctru.leeds.ac.uk/codifi2

We would be grateful if you could also return the most recent copy of your Antibiotic Diary, even if it is blank.

We really appreciate your answers - no matter how you complete the questionnaire - as they give us the best idea of your quality of life after your first visit. Your responses are important, as we need them to make sure that the results are as reliable as possible and may help to change clinical practice for the better!

Thank you for help with this study. We will contact you again with the last questionnaire booklet in 12 months' time to ask how you are getting on.

Yours sincerely,

Andrea Nelson
Chief Investigator, CODIFI2

dd-mm-yyyy

Mr/Miss/Ms Xxxx Xxxxx
XX Xxxxx
Xxxxx
Xxxx
XXX XXX

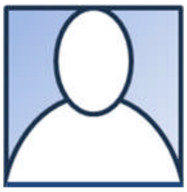

Professor Andrea Nelson
Chief Investigator, CODIFI2

Dear Xxxxx

Concordance in Diabetic Foot Ulcer Infection – 2 (CODIFI2)

I am writing to you to follow up on the CODIFI2 research study that you very kindly agreed to take part in following a visit to <Site Name> around 12 months ago to look at your diabetic foot ulcers. CODIFI2 looks to see whether infected diabetic foot ulcers heal quicker if samples are taken by swabbing or by taking tissue from the affected area. This study is supported by the NHS' National Institute for Healthcare Research and the University of Leeds, and involves hospitals across Britain. If this trial is successful, the results may lead to changes in hospital practices to better manage the diagnosis of people like you, and may lead to better use of antibiotics.

It is now a year since you first agreed to take part in the study, and I am writing to provide the last questionnaire booklet for you to complete. We would now like to know how you are feeling, 12 months after your initial visit. We are keen to hear from you, no matter how your health has changed in this time. To know this, we would like you to complete the enclosed questionnaire, and return it to us in the envelope provided. We would be very grateful if you could complete the questionnaire and return it to us as soon as possible. There are no right or wrong answers to these questions. Your answers will give a broad view of the impact of having a diabetic foot ulcer as time goes on. Neither your GP nor staff at the hospital you attended will be able to see your answers, so you do not need to worry about what they might think of your answers. If you have any questions, please feel free to get in touch with Xxxx Xxxx at the University of Leeds on Xxxx@Leeds.ac.uk. Further information about the study can be found on our website https://ctru.leeds.ac.uk/codifi2

We would be grateful if you could also return the most recent copy of your Antibiotic Diary, even if it is blank.

On behalf of the CODIFI2 trial team, both at <Site Name> and at the University of Leeds, thank you for your help with this study over the past year. We really appreciate your answers - no matter how you complete the questionnaire - as they give us the best idea of your quality of life after your first visit. Your responses are important, as we need them to make sure that the results are as reliable as possible and may help to change clinical practice for the better!

Yours sincerely,

Andrea Nelson
Chief Investigator, CODIFI2

W52 Enhanced Final

W52 Enhanced Final (with thanks)

dd-mm-yyyy

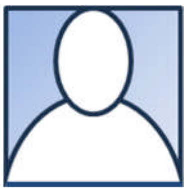

Mr/Miss/Ms Xxxx Xxxxx
XX Xxxxx
Xxxxx
Xxxx
XXX XXX

Professor Andrea Nelson
Chief Investigator, CODIFI2

Dear Xxxxx

Concordance in Diabetic Foot Ulcer Infection – 2 (CODIFI2)

I am writing to you to follow up on the CODIFI2 research study that you very kindly agreed to take part in following a visit to <Site Name> around 12 months ago to look at your diabetic foot ulcers. CODIFI2 looks to see whether infected diabetic foot ulcers heal quicker if samples are taken by swabbing or by taking tissue from the affected area. This study is supported by the NHS' National Institute for Healthcare Research and the University of Leeds, and involves hospitals across Britain. If this trial is successful, the results may lead to changes in hospital practices to better manage the diagnosis of people like you, and may lead to better use of antibiotics.

Thank you for completing and returning your 9 month questionnaire booklet. It is now a year since you first agreed to take part in the study, and I am writing to provide the last questionnaire booklet for you to complete. We would now like to know how you are feeling, 12 months after your initial visit. We are keen to hear from you, no matter how your health has changed in this time. To know this, we would like you to complete the enclosed questionnaire, and return it to us in the envelope provided. We would be very grateful if you could complete the questionnaire and return it to us as soon as possible. There are no right or wrong answers to these questions. Your answers will give a broad view of the impact of having a diabetic foot ulcer as time goes on. Neither your GP nor staff at the hospital you attended will be able to see your answers, so you do not need to worry about what they might think of your answers. If you have any questions, please feel free to get in touch with Xxxx Xxxx at the University of Leeds on Xxxx@Leeds.ac.uk. Further information about the study can be found on our website https://ctru.leeds.ac.uk/codifi2

We would be grateful if you could also return the most recent copy of your Antibiotic Diary, even if it is blank.

On behalf of the CODIFI2 trial team, both at <Site Name> and at the University of Leeds, thank you for your help with this study over the past year. We really appreciate your answers - no matter how you complete the questionnaire - as they give us the best idea of your quality of life after your first visit. Your responses are important, as we need them to make sure that the results are as reliable as possible and may help to change clinical practice for the better!

Yours sincerely,

Andrea Nelson
Chief Investigator, CODIFI2

W52 Enhanced Final (with thanks)

W104 Standard

dd-mm-yyyy

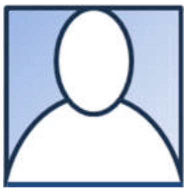

Mr/Miss/Ms Xxxx Xxxxx
XX Xxxx
Xxxxx
XXX XXX

Professor Andrea Nelson
Chief Investigator, CODIFI2

Dear Xxxx

Concordance in Diabetic Foot Ulcer Infection – 2 (CODIFI2)

I am writing to you to follow up on a research study that you very kindly agreed to take part in when you visited <Site Name> around 2 years ago. This study is called CODIFI2, and looks to see whether infected diabetic foot ulcers heal quicker if samples are taken by swabbing or taking tissue from the affected area.

Following your initial visit, you agreed to complete some follow-up questionnaires in the following months. Please take time to complete this questionnaire and return in the provided stamped-addressed envelope. This is the last questionnaire for this study.

We would be grateful if you could also return the most recent copy of your Antibiotic Diary, even if it is blank.

If you have any queries about the questionnaire please don't hesitate to contact Xxxx Xxxx at the University of Leeds on Xxxx@Leeds.ac.uk. Further information about the study can be found on our website https://ctru.leeds.ac.uk/codifi2

Thank you for your help with this study.

Yours sincerely,

Andrea Nelson
Chief Investigator, CODIFI2

W104 Standard

W104 Enhanced

dd-mm-yyyy

Mr/Miss/Ms Xxxx Xxxxx
XX Xxxx Xxxx
Xxxx
Xxxx
XXX XXX

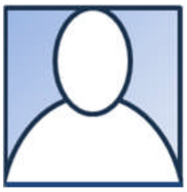

Professor Andrea Nelson
Chief Investigator, CODIFI2

Dear Xxxxx

Concordance in Diabetic Foot Ulcer Infection – 2 (CODIFI2)

I am writing to you to follow up on the CODIFI2 research study that you very kindly agreed to take part in following a visit to <Site Name> around 2 years ago to look at your diabetic foot ulcers. CODIFI2 looks to see whether infected diabetic foot ulcers heal quicker if samples are taken by swabbing or by taking tissue from the affected area. This study is supported by the NHS' National Institute for Healthcare Research and the University of Leeds, and involves hospitals across Britain. If this trial is successful, the results may lead to changes in hospital practices to better manage the diagnosis of people like you, and may lead to better use of antibiotics.

It is now 2 years since you first agreed to take part in the study, and I am writing to provide the last questionnaire booklet for you to complete. We would like to know how you are feeling after your initial visit 2 years ago. We are keen to hear from you, no matter how your health has changed in this time. To know this, we would like you to complete the enclosed questionnaire, and return it to us in the envelope provided. We would be very grateful if you could complete the questionnaire and return it to us as soon as possible. There are no right or wrong answers to these questions. Your answers will give a broad view of the impact of having a diabetic foot ulcer as time goes on. Neither your GP nor staff at the hospital you attended will be able to see your answers, so you do not need to worry about what they might think of your answers. If you have any questions, please feel free to get in touch with Xxxx Xxxx at the University of Leeds on Xxxx@Leeds.ac.uk. Further information about the study can be found on our website https://ctru.leeds.ac.uk/codifi2

We would be grateful if you could also return the most recent copy of your Antibiotic Diary, even if it is blank.

On behalf of the CODIFI2 trial team, both at <Site Name> and at the University of Leeds, thank you for your help with this study over the past 2 years. We really appreciate your answers - no matter how you complete the questionnaire - as they give us the best idea of your quality of life after your first visit. Your responses are important, as we need them to make sure that the results are as reliable as possible and may help to change clinical practice for the better!

Yours sincerely,

Andrea Nelson
Chief Investigator, CODIFI2

W104 Enhanced

Clinical Trials Research Unit
Leeds Institute of Clinical Trials Research
University of Leeds
Leeds
LS2 9JT
http://medhealth.leeds.ac.uk/LICTR

Reminder Letter

Mr/Miss/Ms Xxxxx Xxxxx
XXX Xxxxx Xxxxx
Xxxxxx
XXX XXX

dd-mm-yyyy

Dear Xxxxxx

We recently sent you a set of questionnaires as part of the above study in which you are participating. At the time of writing this letter we have not yet received your completed questionnaires, and so we are writing to remind you about them.

We would be very grateful if you could complete and return them as soon as possible as your experiences are very important to help us understand the effect a diabetic foot ulcer has on people's quality of life. Please be assured your responses will be entirely confidential and will not be seen by any of the people dealing with your treatment.

If you feel you would rather not complete any more questionnaires then please return the blank forms in the stamped addressed envelope provided earlier. If you have lost either the questionnaires or the envelope, or if you feel you would like to discuss the study in more detail, please feel free to contact the CODIFI 2 Data Manager Xxxxx Xxxxx on Xxxx@leeds.ac.uk.

If you have returned the questionnaires to us in the last 7 days please do not respond to this letter.

Many thanks for your time, your help with this study is greatly appreciated.

Yours sincerely

CODIFI 2
CTRU Research Team

Reminder Letter
